# Association between organophosphorus insecticides exposure and osteoarthritis in patients with arteriosclerotic cardiovascular disease

**DOI:** 10.1186/s12889-024-19414-9

**Published:** 2024-07-14

**Authors:** Shenhao Zhu, Yang Zhou, Menglin Chao, Yuqing Zhang, Weili Cheng, Hongyao Xu, Lai Zhang, Qin Tao, Qiang Da

**Affiliations:** 1 Department of Urology, The First Affiliated Hospital of Nanjing Medical University, No 300 Guangzhou Road, Nanjing, 210029 China; 2 Department of Sports Medicine and Joint Surgery, Nanjing First Hospital, Nanjing Medical University, Nanjing, 210006 China; 3 Department of Endocrinology, The First Affiliated Hospital of Nanjing Medical University, No 300 Guangzhou Road, Nanjing, 210029 China; 4 Department of Cardiology, The Affiliated Jiangning Hospital with Nanjing Medical University, Nanjing, 211100 China; 5 Department of Pharmacy, The First Affiliated Hospital of Nanjing Medical University, No 300 Guangzhou Road, Nanjing, 210029 China

**Keywords:** Arteriosclerotic cardiovascular disease, Organophosphorus insecticides, Osteoarthritis, Dose-response relationship, NHANES, BKMR

## Abstract

**Background:**

Organic phosphorus insecticides (OPPs) are a class of environmental pollutants widely used worldwide with potential human health risks. We aimed to assess the association between exposure to OPPs and osteoarthritis (OA) particularly in participants with atherosclerotic cardiovascular disease (ASCVD).

**Methods:**

Participants’ information was obtained from data in the National Health and Nutrition Examination (NHANES). Weighted logistic regression models were utilized to detect associations between OPPs metabolites and OA. Restricted cubic spline plots (RCS) were drawn to visualize the dose-response relationship between each metabolite and OA prevalence. Weighted quantile sum (WQS) regression and Bayesian kernel-machine regression (BKMR), were applied to investigate the joint effect of mixtures of OPPs on OA.

**Results:**

A total of 6871 samples were included in our study, no significant associations between OPPs exposure and OA incidence were found in whole population. However, in a subset of 475 individuals with ASCVD, significant associations between DMP (odds ratio [OR] as a continuous variable = 1.22, 95% confidence interval [CI]: 1.07,1.28), DEP ((odds ratio [OR] of the highest tertile compared to the lowest = 2.43, 95% confidence interval [CI]: 1.21,4.86), and OA were observed. DMP and DEP showed an increasing dose-response relationship to the prevalence of OA, while DMTP, DETP, DMDTP and DEDTP showed a nonlinear relationship. Multi-contamination modeling revealed a 1.34-fold (95% confidence intervals:0.80, 2.26) higher prevalence of OA in participants with high co-exposure to OPPs compared to those with low co-exposure, with a preponderant weighting (0.87) for the dimethyl dialkyl phosphate metabolites (DMAPs). The BKMR also showed that co-exposure of mixed OPPs was associated with an increased prevalence of OA, with DMP showing a significant dose-response relationship.

**Conclusion:**

High levels of urine dialkyl phosphate metabolites (DAP) of multiple OPPs are associated with an increased prevalence of OA in patients with ASCVD, suggesting the need to prevent exposure to OPPs in ASCVD patients to avoid triggering OA and further avoid the occurrence of cardiovascular events caused by OA.

**Supplementary Information:**

The online version contains supplementary material available at 10.1186/s12889-024-19414-9.

## Introduction

Pesticides are widely used in environments such as crop cultivation, horticulture, and household life because of their ability to prevent pathogenic bacterial infections and kill pests [1]. Organophosphorus insecticides (OPPs) composed about 33% of all pesticide applications in the United States due to their low cost and high efficiency [2]. Especially due to the need to control pests that can spread diseases, these pesticides have been applied in the field of public health, increasing exposure in the population [3]. The large amount of OPPs remaining in food, drinking water, soil, and air can enter the human body through oral, skin, inhalation, and eye contact, causing health hazards [4]. Most OPPs are composed of phosphates (or thiophosphates or thiophosphates) and an organic group. In most cases, these phosphates (or thiophosphates or thiophosphates) are replaced by O, O-dialkyl groups, where the alkyl group is usually dimethyl or diethyl. Once inside the body, OPPs can be converted into oxygenated forms by enzymes and then react with available cholinesterase enzymes. The oxon may undergo either enzymatic or spontaneous hydrolysis to produce of dimethyl dialkyl phosphate metabolites (DMAPs) or diethyl dialkyl phosphate metabolites (DEAPs) and organic group moieties. These metabolites and/or their glucuronate or sulfate conjugates are excreted in the urine [5]. Quantification of exposure to OPPs is critical as exposure to OPPs typically occurs through multiple pathways. Measurement of six common DAP metabolites in urine can provide information on exposure to OPPs or exposure to DAP itself, which may be present in the environment as breakdown products of OPPs (environmental DAP), which is the most commonly used quantification method for assessing exposure to OPPs [6]. Mechanistically speaking, OPPs can cause acute and chronic toxicity by inhibiting acetylcholinesterase activity or cholinesterase independent pathways (inducing protein cross-linking into high molecular weight aggregates) [7]. Since the last century, evidence on the neurotoxicity of OPPs has gradually been confirmed and increasingly reported. Growth and neurodevelopmental limitations in children, as well as cognitive impairment and neurodegenerative diseases in adults, such as Parkinson’s disease and Alzheimer’s disease, are all associated with chronic exposure to OPPs [8, 9]. Recent studies have shown that OPPs can also induce diseases beyond the nervous system, including metabolic diseases (obesity and type 2 diabetes) [10], asthma [11], hearing impairment [12], non-Hodgkin’s lymphoma [13], immune response suppression [14] and acute nephrotoxicity [15].

Osteoarthritis (OA), a degenerative disease characterized by loss of cartilage and changes in joint structure, is estimated to afflict 7.6% of the world’s population (595 million people) in 2020, and is expected to torment 1 billion individuals by 2050, posing a significant burden on public health [16]. As the most common type of arthritis, the incidence of OA has nearly doubled in the industrial era and continues to increase. This suggests that in addition to classic risk factors such as obesity and aging, environmental factors play a significant role in the occurrence of osteoarthritis. Epidemiologic studies suggest that polychlorinated biphenyls (PCBs), perfluoroalkyls (PFAS), lead, and other heavy metals are possibly associated with OA [17]. However, there are no large population-based studies on the association of OPPs metabolites with OA, except for a single-center descriptive study that included only 25 patients with OA, which reported that OPPs were detectable in more than 80% of patient serum samples and were the most prominent of all pesticides [18].

Arteriosclerotic cardiovascular disease (ASCVD) most commonly referred to either coronary artery disease, peripheral artery disease, cerebrovascular disease, or aortic atherosclerosis, with plaques forming in any number of vascular beds [19]. ASCVD has been the leading cause of mortality in developed countries since the mid-20th century, imposing a huge burden on modern societies and becoming a growing public health problem [20]. The comorbidities (e.g., chronic kidney disease, hypertension, diabetes mellitus, dyslipidemia, etc.) of ASCVD are also important risk factors for cardiovascular disease, and these disorders create a mutually reinforcing positive feedback loop of endothelial inflammation, injury, and dysfunction, ultimately delivering multiple hits to the patient [21]. Therefore, prevention and treatment of comorbidities in patients with ASCVD is essential to reduce cardiovascular-caused mortality. Patients with cardiovascular disease often have some genetic changes, which are not only crucial to the development of atherosclerosis, but also participate in the detoxification process of OPPs [22–26]. Therefore, cardiovascular disease patients may be more sensitive to exposure to OPPs, and studying the relationship between pesticide exposure and arthritis occurrence in this population can help evaluate comorbidity risk and provide patients with more comprehensive health management. Reducing pesticide exposure may help improve the quality of life of cardiovascular disease patients and reduce pain and discomfort caused by arthritis. However, no current studies have focused on the relationship between OPPs exposure and OA in a population of patients with cardiovascular disease. Our study aimed firstly to address the issue of whether exposure to OPPs is associated with suffering OA, and secondly to investigate whether OPPs are associated with concomitant OA in ASCVD, a group of patients prone to comorbidities, through publicly available large-scale survey data, and thus provide epidemiologic evidence for public health decision-making.

## Materials and methods

### Data source and study population

NHANES is a continuous, cross-sectional survey conducted by the Centers for Disease Control and Prevention that combines interviews and physical examinations to study the health and nutritional status of US population. Using a complex, multistage, stratified, clustered probability sampling design, NHANES collects sample data every 2 years to represent the entire population of the United States. A random one-third subsample of all survey participants in eight 2-year cycles from 1999 to 2018 (except 2009 to 2010) had urinary OPPs metabolite measurements and were included in this study.

### Measurements of urinary organophosphorus insecticides metabolites

Six measurable dialkyl phosphate (DAP) metabolites were considered non-specific biomarkers of exposure to at least 28 OPPs, including dimethylphosphate (DMP); dimethylthiophosphate (DMTP); dimethyldithiophosphate (DMDTP); diethylphosphate (DEP); diethylthiophosphate (DETP); and diethyldithiophosphate (DEDTP). These metabolites were quantitatively measured by freeze-drying and chemical derivatization of urine specimens followed by isotope-dilution gas chromatography-tandem mass spectrometry (GC-MS/MS) analysis. Urine samples were processed and analyzed according to the method of Bravo et al. [6]. Specifically, 4 milliliters of urine were added to an isotopic labeled internal standard mixture and then concentrated to dryness using azeotropic distillation with acetonitrile. The residue is dissolved in acetonitrile, and dialkyl phosphates (DAPs) are derived into their respective chloropropyl esters using 1-chloro-3-iodopropane and potassium carbonate. The solution containing chloropropyl ester was concentrated and analyzed using gas chromatography-positive chemical ionization-tandem mass spectrometry. Quantification of DAP metabolites using isotope-dilution calibration method. The urine creatinine value of each sample was extracted and used to correct for the concentration of DAPs. The details of laboratory measurements can be found in a previous literature.

### Assessment of ASCVD

ASCVD assessment was obtained from the Medical Conditions questionnaire, which was self- and proxy-reported by participants on a broad range of health conditions and medical history for both children and adults. The NHANES MCQ section is generally modeled on the “Medical Conditions” questionnaire section of the U.S. National Health Interview Survey.

The following 4 questions answered by participants are the basis for our assessment:

1. “Ever told you had coronary heart disease?”
2. “Ever told you had angina/angina pectoris?”
3. “Ever told you had heart attack?”.
4. “Ever told you had a stroke?”.

Participants who answered “yes” to one of the questions were considered as ASCVD group. The remaining participants were classified as nonregular users who answered “No” to the second question and those who had never ASCVD.”

### Assessment of arthritis

The NHANES medical conditions (variable name prefix MCQ) section provides self- and proxy-reported personal interview data on the conditions of arthritis, including Osteoarthritis or degenerative arthritis (OA), Rheumatoid arthritis (RA), Psoriatic arthritis and others.

The following 2 questions were used to obtain the morbidity of arthritis in participants :

1. “Doctor ever said you had arthritis”.
2. “Which type of arthritis was it?”

Participants who answered “yes” to questions 1 were considered as who had Arthritis. The remaining participants were classified as no Arthritis group.” Furthermore, the Arthritis group was classified as OA, RA, and others.

### Other covariates

#### Demographic variables

Individuals’ self-reported age, sex, race, annual household income, family income-to-poverty ratio, education level, and marital status at interview were obtained from the NHANES demographic files.

#### Body mass index

Body mass index data of study subjects were recorded in NHANES body measurements, which were collected by trained health technicians in the MEC.

### High-density lipoprotein (HDL)& triglycerides& total cholesterol

The HDL-C concentration、Triglycerides concentration and Total cholesterol concentration were calculated from the blood lipids measurements in NHANES survey include total cholesterol, high-density lipoprotein cholesterol (HDL-C), low-density lipoproteins cholesterol (LDL-C), and triglycerides. Data on total cholesterol are provided in Cholesterol - Total (TCHOL_J) file, and HDL-C data are provided in Cholesterol - High - Density Lipoprotein (HDL_J).

#### Tobacco use

Smoking status was constructed from responses to 2 items from the questionnaire section : 1) “Have you smoked at least 100 cigarettes in your entire life?” and 2) “Do you now smoke cigarettes?” Respondents who reported smoking every day or some days and had smoked > 100 cigarettes were categorized as “current smokers”; respondents who reported currently not smoking but having smoked > 100 cigarettes in the past were categorized as former smokers; and respondents who reported having smoked < 100 cigarettes ever were categorized as nonsmokers.

#### Alcohol use

Alcohol use data were collected during the MEC interview. A drink was defined as a “12 oz. beer, 5 oz. glass of wine, or one and a half ounces of liquor”. Participants were asked if they had consumed at least 12 drinks in their entire life and in any one year. They were also asked, “In the past 12 months,”: (1) “how often did you drink any type of alcoholic beverage?” (drinking frequency – days per week, month, or year); and (2) “on those days that you drank alcoholic beverages, on the average, how many drinks did you have?” .In this paper, the specific classification criteria of alcohol use refer to the methods in previous literature [27, 28].

#### Estimated glomerular filtration rate

We calculated estimated glomerular filtration (eGFR) rate using the Chronic Kidney Disease Epidemiology Collaboration equation, as reported in a previous study [29].

#### Diabetes mellitus

A subject meeting any of the following criteria will be diagnosed with diabetes [18]: (1) Doctor told you that you have diabetes; (2) glycohemoglobin HbA1c(%) > 6.5; (3) fasting glucose (mmol/L) ≥ 7.0; (4) random blood glucose (mmol/L) ≥ 11.1; (5) 2-hour Oral Glucose Tolerance Test (OGTT) blood glucose (mmol/L) ≥ 11.1; 6)use of antidiabetic agents.

#### Hypertension

Hypertension in NHANES was defined as participant self-reported hypertension (answer “yes” to the question “Have you ever been told by a doctor or other health professional that you had hypertension, also called high blood pressure?”) or elevated blood pressure during physical examination (mean systolic blood pressure ≥ 140 mm Hg, or mean diastolic blood pressure ≥ 90 mmHg).

#### Physical activity

Physical activity was collected from the Physical Activity questionnaire (PAQ) in NHANES. PAQ was asked in the home, by trained interviewers, using the Computer Assisted Personal Interview (CAPI) system. Different types of physical activity have different MET (Metabolic equivalent) values, and NHANES provides recommended MET values for different types of exercise. The PAQ survey including Vigorous work-related activity (MET = 8), Moderate work-related activity (MET = 4), Walking or bicycling for Transportation (MET = 4), Vigorous leisure-time physical activity (MET = 8) and Moderate leisure-time physical activity (MET = 4). PA can be calculated based on the MET value, activity type, weekly frequency, and duration. We calculated the value of PA according to the following formula: PA (MET-h/wk) = MET × weekly frequency × duration of each physical activity [30].

All adjustment variables and their details are summarized presented in Table S1.

### Data Analysis

We utilized the R software (version 4.1.2, Bell Laboratories) for all statistical procedures. Due to the complex sampling design of NHANES, we considered strata, primary sampling unit, and sampling weights in our data analysis lined with the National Center for Health Statistics directions to estimate statistics for the entire US population. Creatinine-corrected concentrations of urinary OPPs metabolites were used for further analysis after logarithmic transformation to a normally distributed distribution.

According to the self-reported medical condition, the included subjects were considered to have one or more diseases (ASCVD, OA, hypertension, diabetes). The presence of OA in the subjects and the concentration of OPPs metabolites were used as outcome variables and exposure variables, respectively, in all analysis processes. All subjects were divided into two subgroups (non ASCVD and ASCVD) based on whether they had ASCVD. Statistical differences between cases with OA and non-OA were evaluated for categorical and continuous variables using chi-square and t tests, respectively. Multivariate survey-weighted logistic regressions adjusted for those covariates that were significantly different between the two groups (OA patients and non-OA) were used to assess the association of OPPs exposure with prevalence of OA in whole populations or subsets of included samples. Concentrations of OPPs metabolites were used as continuous and categorical variables (divided into tertiles) to calculate the odds ratio of OA individually.

In the subsets with ASCVD, subgroup analyses were performed to ascertain whether the association varied across gender, age group, and ethnicity. Nonlinear relationships between levels of various OPPs metabolites and overall OA events were tested by drawing restricted cubic spline (RCS) curves with three knots. Furthermore, we used a weighted quantile sum (WQS) regression, which is widely used in the field of environmental epidemiology [31], to determine the health effects of mixed exposures to these six metabolites of OPPs and the relative weights of single chemical, based on methodologies described in previous literature [32]. Another novel statistical method, Bayesian kernel-machine regression (BKMR), was also used to assess the exposure-response relationship for a mixture of chemicals [33, 34], which addressed two issues: (1) the effect of individual OPPs metabolites on OA when the concentration of other OPPs metabolites was fixed at the median; and (2) the overall effect of OPPs metabolites mixture at different percentile on OA, compared to when all OPPs metabolites were kept at the 50th percentile.

## Results

Of the 111,797 participants, 64,165 individuals were included in our primary selection after removing participants with missing OA and ASCVD prevalence information. From these, we further screened a subsample of 12,793 participants registered for urine DAP metabolite testing, and after excluding records with missing values for covariates (*n* = 5651) and missing sample weights (*n* = 271), 6871 subjects were included in our final analysis, of whom 475 had ASCVD (Figure S1 shows the flowchart of sample selection).

Patients with ASCVD were older, had a higher BMI, were less physically active, had a predominant history of smoking and alcohol consumption, had elevated ALP and GGT values, had elevated triglycerides and decreased total cholesterol and HDL, and were more often associated with hypertension and diabetes compared to the non-ASCVD population (Table S2). In the non-ASCVD patient population as well as in the overall population, multivariate logistic regression results did not reveal a significant association of these six DAP metabolites with OA (Table S3).

These ASCVD patients have an average age of 61.5 years, with 305 males and 170 females, of which over 80% are overweight (30 kg/m^2^ > BMI ≥ 25 kg/m^2^) or obese (BMI ≥ 30 kg/m^2^). Proportionality of coronary heart disease, angina, myocardial infarction, and stroke., are 47.09%, 33.74%, 44.47%, and 36.00%, respectively. Table 1 and Table S4 summarizes the weighted and unweighted baseline characteristics of the included subjects, respectively. The majority of the selected subjects were non-Hispanic white populations (56.63%), male (64.21%), with a high school education or above (74.31%), and had a history of smoking and alcohol use. Among these ASCVD patients, 76.42% were accompanied by hypertension and 33.68% were accompanied by diabetes. Among these ASCVD patients, 76.42% were accompanied by hypertension and 33.68% were accompanied by diabetes. The highest median concentrations of these OPPs metabolites in the urine samples of the subjects were DMTP, DMP, and DEP, with lower exposure levels for the remaining three metabolites. Of particular note, we can see from Table 1 that 23.01% of ASCVD patients also have OA. Compared to non OA patients, OA patients tend to be elderly, female, with low bilirubin levels, and low eGFR. There were no significant differences in the concentrations of various OPPs metabolites and other covariates between the two groups.

**Table 1:** Weighted basic characteristic of the participants with ASCVD

variable	total (weighted *N* = 7,190,068)	non-OA (weighted *N* = 5,535,380)	OA (weighted *N* = 1,654,688)	P value
Age in years, n(%)				**0.01**
20-34	16( 5.02)	15(6.45)	1(0.25)	
35-64	210(46.81)	176(50.15)	34(35.61)	
>=65	249(48.17)	190(43.39)	59(64.15)	
Sex, n(%)				**< 0.001**
Female	170(35.65)	115(29.10)	55(57.55)	
Male	305(64.35)	266(70.90)	39(42.45)	
Race/Ethnicity, n(%)				0.25
white	269(76.76)	202(75.08)	67(82.37)	
black	89( 8.05)	76(8.87)	13(5.32)	
mexican	51( 3.51)	49(4.35)	2(0.72)	
other	66(11.67)	54(11.70)	12(11.59)	
Education, n(%)				0.19
< high school	122(16.15)	105(18.12)	17( 9.57)	
high school	116(25.84)	91(24.79)	25(29.35)	
> high school	237(58.01)	185(57.09)	52(61.08)	
Alcohol use, n(%)				0.51
never	62(11.21)	47(10.97)	15(12.01)	
former	117(22.16)	96(22.49)	21(21.04)	
mild	195(43.87)	149(41.50)	46(51.83)	
moderate	46( 9.13)	39(10.31)	7( 5.16)	
heavy	55(13.64)	50(14.74)	5( 9.96)	
Smoke, n(%)				0.35
never	179(37.43)	137(35.85)	42(42.70)	
former	202(42.17)	166(41.84)	36(43.29)	
now	94(20.40)	78(22.31)	16(14.01)	
Hypertension, n(%)	363(74.58)	286(72.53)	77(81.41)	0.13
Diabetes mellitus, n(%)	160(30.67)	131(30.23)	29(32.14)	0.93
Physical activity, n(%)*				0.51
<600	161(34.44)	123(33.59)	38(37.26)	
600-7999	274(58.07)	225(59.61)	49(52.89)	
>=8000	40( 7.50)	33(6.79)	7(9.85)	
BMI (kg/m^2^),mean±SE	30.14±0.34	30.33±0.41	29.50±0.56	0.22
HbA1c (%), mean±SE	6.01±0.07	6.01±0.08	5.98±0.15	0.87
ALT (u/l), mean±SE	26.68±1.61	26.94±1.93	25.82±2.25	0.7
AST (u/l), mean±SE	25.86±0.92	25.97±1.11	25.47±1.48	0.79
Bilirubin (mg/dl), mean±SE	0.70±0.02	0.72±0.02	0.63±0.03	**0.01**
ALP (u/l), mean±SE	74.26±1.53	74.72±1.87	72.73±1.89	0.44
GGT (u/l), mean±SE	33.33±2.65	34.80±3.38	28.44±2.44	0.14
Triglycerides (mmol/l), mean±SE	1.90±0.07	1.86±0.09	2.02±0.11	0.27
Cholesterol (mmol/l), mean±SE	4.79±0.07	4.76±0.08	4.88±0.15	0.5
HDL (mmol/l), mean±SE	1.28±0.03	1.27±0.04	1.34±0.04	0.22
eGFR (mL/min/1.73 m2), mean±SE	79.20±1.39	80.53±1.53	74.77±2.28	**0.03**
DEP (μg/L), median [IQR]	1.34[0.26,4.25]	1.17[0.26,3.69]	1.88[0.38,6.53]	0.24
DMP (μg/L), median [IQR]	1.34[0.35,4.41]	1.21[0.35,3.97]	2.34[0.75,5.41]	0.18
DMTP (μg/L), median [IQR]	1.61[0.39,5.65]	1.58[0.39,5.62]	2.02[0.57,6.09]	0.32
DETP (μg/L), median [IQR]	0.40[0.10,0.63]	0.40[0.14,0.63]	0.25[0.07,0.73]	0.71
DMDTP (μg/L), median [IQR]	0.22[0.07,0.46]	0.23[0.07,0.53]	0.22[0.07,0.42]	0.67
DEDTP (μg/L), median [IQR]	0.07[0.07,0.28]	0.07[0.07,0.28]	0.07[0.07,0.07]	0.61
DEP (ng/g creatinine), median [IQR]	15.32[3.36,39.83]	13.99[2.84,33.52]	24.23[8.48,42.80]	0.17
DMP (ng/g creatinine), median [IQR]	14.90[4.49,34.62]	13.02[3.93,32.85]	22.07[7.75,77.24]	0.11
DMTP (ng/g creatinine), median [IQR]	18.53[4.21,51.41]	17.03[4.21,42.68]	27.52[6.00,94.38]	0.36
DETP (ng/g creatinine), median [IQR]	3.38[1.26,7.14]	3.44[1.40,7.07]	2.75[0.91,7.50]	0.55
DMDTP (ng/g creatinine), median [IQR]	2.35[0.72,7.07]	2.13[0.73,6.56]	2.48[0.67,7.46]	0.47
DEDTP (ng/g creatinine), median [IQR]	0.89[0.47,2.20]	0.92[0.49,2.20]	0.83[0.43,2.19]	0.66

ASCVD: atherosclerotic cardiovascular disease *according to metabolic equivalent (MET) scores OA: osteoarthritis BMI: body mass index HbA1c: glycohemoglobin ALT: alanine aminotransferase AST: aspartate aminotransferase ALP: alkaline phosphatase GGT: gamma glutamyl transferase HDL: high density lipoprotein eGFR: Estimated glomerular filtration rate DEP: diethylphosphate DMP: dimethylphosphate DMTP: dimethylthiophosphate DETP: diethylthiophosphate DMDTP: dimethyldithiophosphate DEDTP: diethyldithiophosphate

Each OPPs metabolite concentration was treated as a continuous and categorical variable, respectively, and its relationship with OA occurrence was presented in Table 2. In the crude model, the incidence of OA increased continuously with higher concentrations of DEP and DMP, both as continuous and tertiary categorical variables. In the fully adjusted model, only the concentration of DMP was independently associated with prevalence of OA. An increase in the natural logarithmic units of DMP was associated with an increased prevalence of OA (odds ratio [OR] = 1.22, 95% confidence interval [CI]: 1.07,1.28). When categorized by tertiles, intermediate to high levels of DMP exposure were associated with an elevated prevalence of OA, although this was only statistically significant in the intermediate group (middle tertile compared with lowest tertile, odds ratio [OR] = 2.94, 95% confidence interval [CI]: 1.42,6.09 ). Model 2, adjusted only for age and sex, led to the same conclusion. Unexpectedly, in either model, the prevalence of OA was lower in the moderately DETP-exposed population compared with the lowly exposed population.

**Table 2:** Association between OPPs exposure and OA among ASCVD patients

	ln-transformed (continuous variables)	Tertile 1 OR (95% CI)	Tertile 2 OR (95% CI)	Tertile 3 OR (95% CI)	*P* for trend
DEP		[-8.86,-5.30]	(-5.30,-3.68]	(-3.68,0.49]	
Model 1	**1.27(1.10**,**1.48)**	reference	1.82(0.85,3.87)	**2.43(1.21**,**4.86)**	**0.009**
Model 2	1.14(0.96,1.34)	reference	1.50(0.68,3.31)	1.62(0.78,3.36)	0.194
Model 3	1.11(0.94,1.32)	reference	1.36(0.62,3.00)	1.49(0.70,3.17)	0.298
DMP		[-8.78,-5.16]	(-5.16,-3.66]	(-3.66,1.90]	
Model 1	**1.33(1.13**,**1.56)**	reference	**3.56(1.81**,**6.99)**	**2.95(1.40**,**6.20)**	**0.005**
Model 2	**1.20(1.00**,**1.45)**	reference	**3.13(1.54**,**6.34)**	2.14(0.97,4.72)	0.098
Model 3	**1.22(1.01**,**1.46)**	reference	**2.94(1.42**,**6.09)**	2.15(0.97,4.76)	0.085
DMTP		[-8.46,-5.10]	(-5.10,-3.66]	(-3.66,3.17]	
Model 1	1.15(0.99,1.34)	reference	0.73(0.39,1.34)	1.82(1.03,3.22)	0.033
Model 2	1.08(0.90,1.28)	reference	0.60(0.30,1.19)	1.27(0.64,2.49)	0.341
Model 3	1.11(0.93,1.32)	reference	0.59(0.30,1.17)	1.41(0.73,2.73)	0.211
DETP		[-9.06,-6.27]	(-6.27,-5.17]	(-5.17,1.04]	
Model 1	0.91(0.70,1.19)	reference	**0.50(0.25**,**0.98)**	0.91(0.50,1.65)	0.674
Model 2	0.81(0.63,1.04)	reference	**0.44(0.20**,**0.93)**	0.66(0.35,1.22)	0.18
Model 3	0.82(0.64,1.05)	reference	**0.44(0.20**,**0.95)**	0.68(0.36,1.27)	0.204
DMDTP		[-9.06,-6.76]	(-6.76,-5.52]	(-5.52,0.43]	
Model 1	1.04(0.90,1.21)	reference	1.20(0.62,2.31)	1.18(0.71,1.96)	0.526
Model 2	0.96(0.80,1.15)	reference	0.85(0.41,1.77)	0.80(0.43,1.48)	0.479
Model 3	1.00(0.83,1.20)	reference	0.85(0.41,1.75)	0.91(0.49,1.69)	0.758
DEDTP		[-9.46,-7.41]	(-7.41,-6.36]	(-6.36,1.01]	
Model 1	1.00(0.76,1.30)	reference	0.70(0.34,1.43)	0.72(0.37,1.40)	0.33
Model 2	0.86(0.63,1.16)	reference	0.57(0.27,1.18)	0.55(0.27,1.10)	0.093
Model 3	0.88(0.64,1.21)	reference	0.57(0.27,1.20)	0.57(0.28,1.18)	0.132

OPPs: organophosphorus insecticides OA: osteoarthritis ASCVD: atherosclerotic cardiovascular disease DEP: diethylphosphate DMP: dimethylphosphate DMTP: dimethylthiophosphate DETP: diethylthiophosphate DMDTP: dimethyldithiophosphate DEDTP: diethyldithiophosphate Model 1 was crude model. Model 2 was further adjusted for sex and age Model 3 further controlled bilirubin and eGFR

The results of the subgroup analyses after stratification by sex, age, and race are presented in Table 3. Compared with the lowest tertile, the odds ratio [OR] of DEP in the highest tertile and DMP in the middle tertile for OA among participants older than 60 years was 2.76 (95% confidence interval [CI]: 1.18,6.47) and 3.62 (95% confidence interval [CI]: 1.49,8.82), respectively. In the female population, the odds ratios [OR] of continuous ln-converted DMP as well as intermediate tertile DMP for OA were 1.36 (95% confidence interval [CI]: 1.01,1.82) and 3.39 (95% confidence interval [CI]: 1.50, 7.62), respectively. Among non-Hispanic white populations, the odds ratio [OR] of moderate compared with lowest levels of DMP for OA was 3.61 (95% confidence interval [CI]: 1.55,8.39).

**Table 3:** Subgroup analyses on the weighted relationship between DEP and DMP with the prevalence of OA among ASCVD patients

	subgroup	ln-transformed (continuous variables)	*p* value	Tertile 1 OR (95%CI)	Tertile 2 OR (95%CI)	Tertile 3 OR (95%CI)	*p* for trend
DEP	Female	1.05(0.82,1.34)	0.68	Reference	1.60(0.41,6.18)	1.43(0.47,4.28)	0.572
	Male	1.16(0.87,1.55)	0.31	Reference	1.11(0.35,3.54)	1.50(0.51,4.43)	0.442
	Age < 60	0.92(0.57, 1.47)	0.72	Reference	0.96(0.25, 3.58)	0.36(0.05, 2.62)	0.301
	Age ≥ 60	**1.22(1.01**,**1.48)**	**0.04**	Reference	1.64(0.62,4.37)	**2.76(1.18**,**6.47)**	**0.018**
	White	1.11(0.91,1.36)	0.28	Reference	1.35(0.53,3.46)	1.55(0.66,3.63)	0.295
	Black	1.05(0.60,1.82)	0.85	Reference	0.93(0.12,7.31)	1.22(0.20,7.23)	0.746
	Other races	1.44(0.68,3.04)	0.32	Reference	1.56(0.17,14.02)	2.17(0.23,20.31)	0.569
DMP	Female	**1.36(1.01**,**1.82)**	**0.04**	Reference	**3.39(1.50**,** 7.62)**	3.09(0.79,12.16)	0.141
	Male	1.11(0.85,1.45)	0.44	Reference	2.47(0.79,7.69)	1.58(0.55,4.54)	0.407
	Age < 60	1.18(0.81, 1.74)	0.37	Reference	1.69(0.37, 7.85)	1.62(0.40, 6.58)	0.448
	Age ≥ 60	1.23(1.00,1.52)	0.05	Reference	**3.62(1.49**,**8.82)**	2.59(0.96,7.04)	0.122
	White	1.23(1.00,1.51)	0.05	Reference	**3.61(1.55**,**8.39)**	2.30(0.95,5.58)	0.086
	Black	1.21(0.78,1.89)	0.34	Reference	1.65(0.29, 9.34)	2.66(0.41,17.45)	0.26
	Other races	1.44(0.76,2.74)	0.25	Reference	2.98(0.31,28.64)	3.55(0.55,23.06)	0.327

DEP: diethylphosphate DMP: dimethylphosphate OA: osteoarthritis ASCVD: atherosclerotic cardiovascular disease The model was adjusted for age, sex, bilirubin, and eGFR

**Fig. 1:**
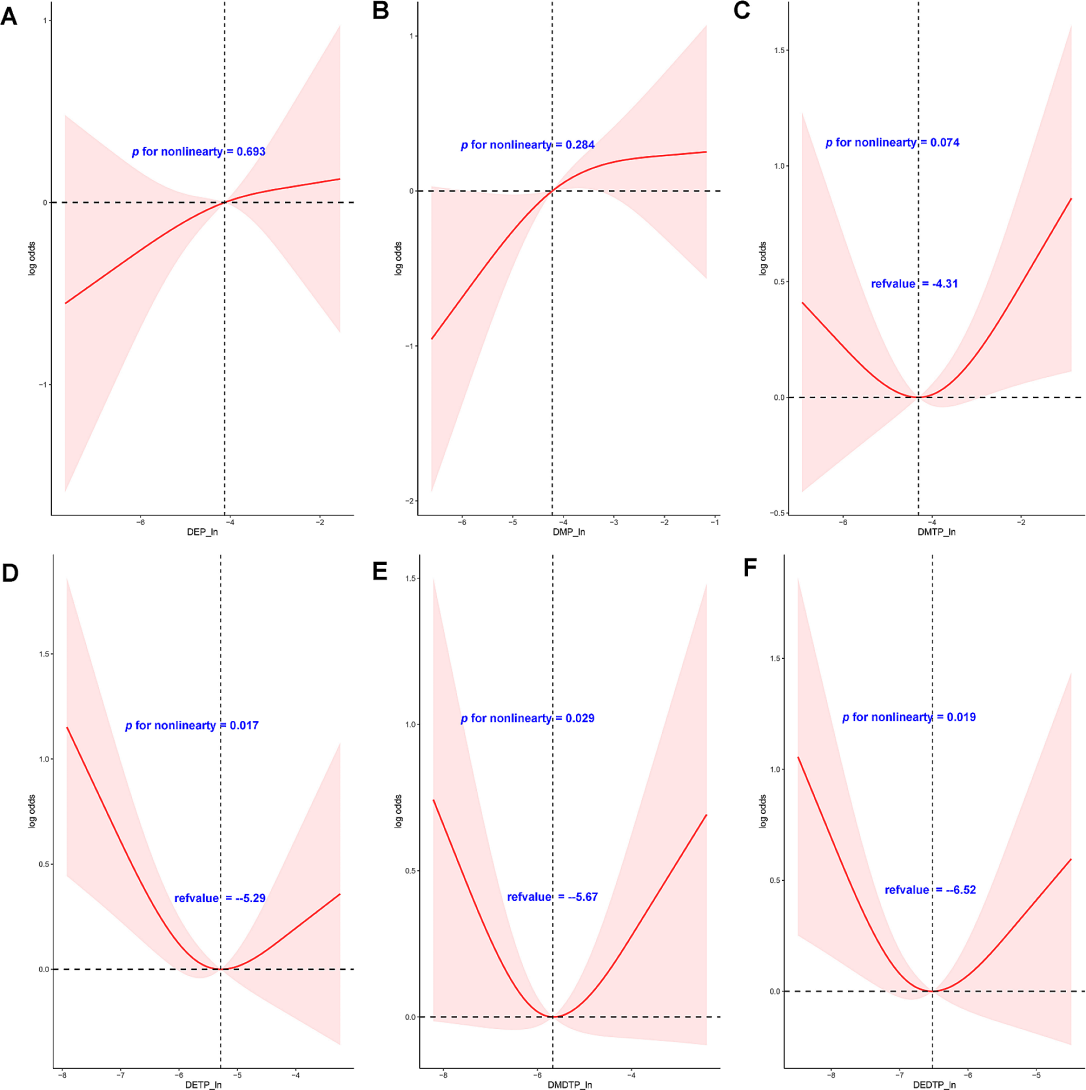
Restricted cubic spline models representing the associations between ln-transformed urinary OPPs metabolite concentrations and osteoarthritis. The model was adjusted for sex, age, bilirubin and eGFR (completely-adjusted model). **A**: DEP; **B**: DMP; **C**: DMTP; **D**: DETP; **E**: DMDTP; **F**: DEDTP

The RCS curves (Fig. 1) resolved the linearly increasing relationship between ln-transformed urinary DEP or DMP concentrations and OA, which was broadly consistent with the results of multivariate logistic regression. In addition, the remaining four OPPs metabolites showed a U-shaped nonlinear relationship, except for DMTP, which was not significant.

**Fig. 2:**
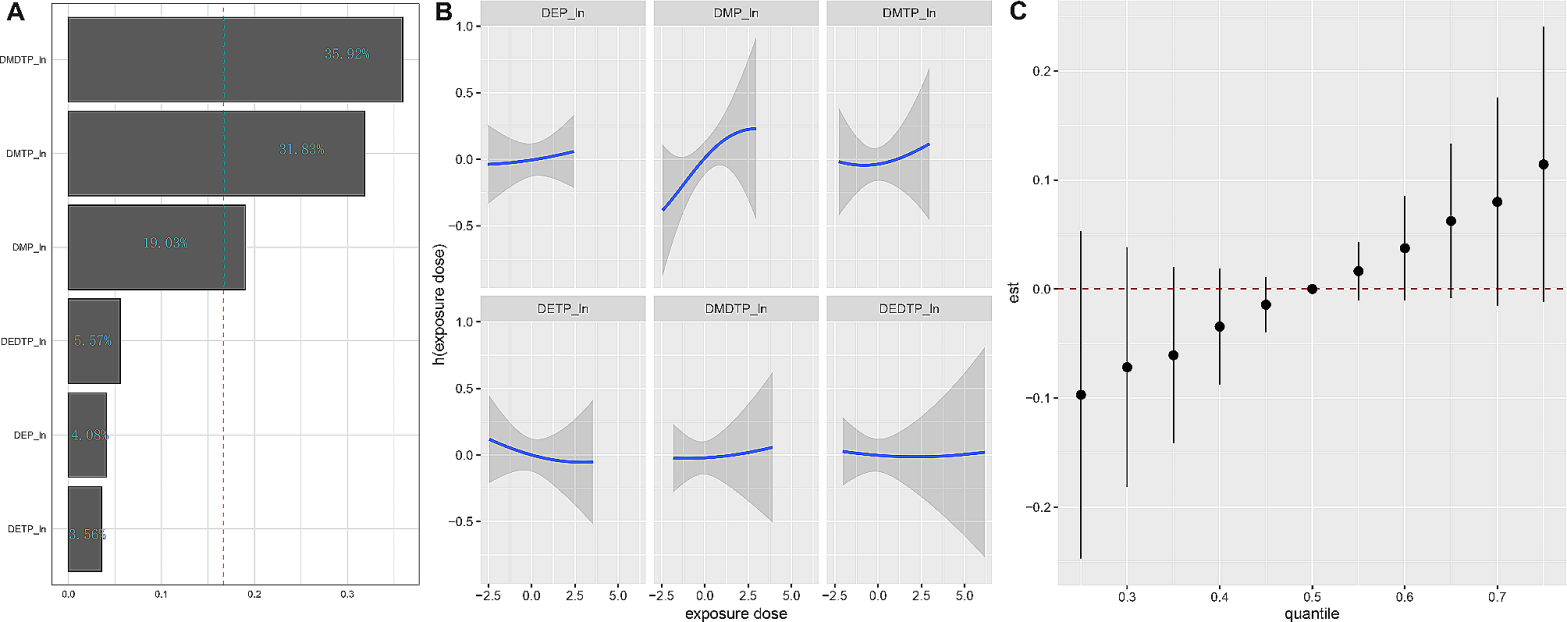
(**A**)The weights of each OPPs metabolite in WQS model regression index for osteoarthritis. (**B**) Dose-response function (95%CI) between each chemical of the OPPs metabolite and osteoarthritis while fixing other chemicals at median values. All OPPs metabolite concentrations were ln-transformed prior to BKMR analysis. (**C**) Overall effect of the OPE mixture on osteoarthritis by BKMR analysis. All models were adjusted for sex, age, bilirubin and eGFR (completely-adjusted model)

Furthermore, two multi-contaminant statistical strategies (WQS regression and BKMR) were used to assess the effect of the OPPs mixture on OA. In the fully adjusted model, the WQS index of the mixture of OPPs was positively associated with an increased prevalence of OA in the overall participants (OR 1.34; 95% CI:0.80, 2.26). DMDTP (35.92%), DMTP (31.83%), and DMP (19.03%) contributed to the WQS index in the top three, followed by DEDTP (5.57%), DEP (4.08%), and DETP (3.56%) (Fig. 2A). The BKMR model illustrated the effect of individual OPP exposure and mixed exposure on OA. We found that exposure to DMP alone was associated with an increased prevalence of OA. The univariate exposure-response curves for DEP, DMTP, DETP, DMDTP, and DEDTP exposure and OA prevalence were relatively flat (Fig. 2B).The cumulative effect of exposure to OPPs was positive, indicating that mixtures of OPPs were positively associated with OA prevalence (Fig. 2C). Although the overall effect of the metabolism of the six OPPs on OA prevalence was not statistically significant when all OPPs metabolites were above their 50th percentile, there was still a clear trend toward an increase.

## Discussion

Our study is the first large-scale cross-sectional investigation of the relationship between OPPs exposure and OA. Our preliminary analyses showed no significant relationship between OPPs exposure and OA prevalence in the whole population and in a subsample of patients without ASCVD (Table S3), and we therefore refocused more on the effect of OPPs on the incidence of OA in patients with ASCVD. DMP and DEP were significantly related to higher prevalence of OA in multivariate regression models, and a linear dose-response relationship was observed. In subsequent subgroup analyses, older adults, females, and non-Hispanic white populations appeared to be more susceptible to the arthrotoxicity of OPPs. Although the correlation between other metabolites is not significant, the RCS curve reveals an increasing trend in the incidence of OA after the concentration of metabolites exceeds a certain threshold. Synergistic exposure to multiple OPPs increased the prevalence of OA in ASCVD patients in multi-pollutant models (WQS and BKMR). Of these, three dimethyl dialkyl phosphate metabolites (DMAPs) contributed the most to the WQS index, implying that the toxicity of these three DMAPs may be more significant, with the toxicity of DMP appearing to be dose-dependent.

We found that the predominant metabolites in the U.S. ASCVD population were DMP, DMTP, DEP, whose urinary concentrations were an order of magnitude higher than those of other DAPs. Despite numerical differences due to heterogeneity of assay methods and biological samples, several population-wide epidemiologic surveys from North America [5, 35], Asia [36], and Europe [37] showed general agreement with the trend of our results. Ye, M. et al. found that DMP, DMTP, and DEP were detected in more than 65% of urine samples from 3,466 Canadian participants aged 20–79 years, with median creatinine-corrected concentrations of 27.5, 14.5, and 17.9 nmol/g, respectively [35]. The main metabolites of OPPs in the Israeli population are DMP and DMTP, whose exposure levels are dozens of times higher than those of adults in the U.S. and Canada attributable to the widespread agricultural application of organic phosphorus pesticides in Israel and the high consumption of fresh and self-produced fruits and vegetables. Notably, analyte concentrations of total DAP (median, 17.6–24.9 µg/g creatinine) were significantly higher in the occupationally exposed population than in the general population [37, 38]. In addition, the concentrations total DAP in the urine of pregnant women in agricultural community and specific urban residential area were much higher than the exposure level among women of childbearing age in general regions [39–41]. The participants in the CHAMACOS birth cohort were pregnant women from the agricultural region of Salinas Valley, California, whose urine levels of DAP metabolites were 2.5 times higher than those of NHANES women, with a median concentration of 112.7 nmol/g creatinine [39]. The Generation R study from Rotterdam, Netherlands reported a higher median total DAP exposure in pregnant women compared to CHAMACOS and NHANES, reaching 316.0 nmol/g creatinine [40]. Further analysis revealed that increased intake of fruits and vegetables in the CHAMCOS and Generation R cohorts were the determining factors for elevated levels of DAP metabolites in pregnant women [42, 43]. Noting that certain populations are more susceptible to the damaging effects of environmental exposure to non-high levels of OPPs due to specific genetic characteristics [44]. [39–41]. Therefore the next studies should emphasize the toxicological effects in individuals susceptible to OPPs. Previous studies have demonstrated that mutations in the paraoxonase (PON) gene family polymorphisms act as protective factors against coronary heart disease, with a 7% decrease in prevalence in patients with coronary heart disease compared to control subjects [45]. PONs are not only involved in preventing atherosclerosis prophylaxis, but also have a wide range of physiological hydrolytic activities, including drug metabolism and detoxification of neurotoxic agents, and play a role in regulating sensitivity to specific pesticides or neurotoxic agents [22, 24]. Other cardiovascular disease-related gene signatures, such as ABCB1 and GST, have also been proposed to be associated with susceptibility to pesticide toxicity [23, 25, 26]. Our study found that compared to non ASCVD patients, only ASCVD patients observed a significant positive correlation between OA occurrence and OPPs exposure at almost the same dose of OPPs exposure levels (Table 2, Table S2, Table S3). The genetic changes in ASCVD patients seem to provide a new research perspective to explain the susceptibility differences of OPPs to joint toxicity, but further research is needed to demonstrate the correlation differences between the two populations.

The stratified analysis results showed that the susceptibility of joints to OPPs exposure is gender dependent. There was no significant difference in exposure levels of OPPs between female ASCVD patients and female ASCVD patients (Table S5), but in female ASCVD patients, OPPs exposure was significantly positively correlated with concurrent OA (Table 3). Similar findings have been made in other studies regarding the gender differences in the toxicity of organophosphorus compounds. Shaffo, Frances C et al. reported that a widely used organophosphorus pesticide, chlorpyrifos, induces airway hyperresponsiveness in female rats at lower doses and rapid response times [46]. Another study of organophosphorus pollutants found that female zebrafish showed more pronounced phenotypic alterations such as intestinal microbiota compared to males despite lower concentrations of 2-ethylhexyl diphenyl phosphate (EHDPHP) [47]. This phenotypic gender difference that occurs at extremely low exposure levels implies that different target distributions of the action of OPPs between genders, as well as different mediating mechanisms, may be a topic worthy of deeper investigation in the future.

Osteoarthritis is a whole joint disease characterized by alterations in the composition, structure, and material properties of single or multiple joint tissues, including periarticular bone, articular cartilage, and synovial bursae, caused by factors of cellular or matrix origin [48]. Little research exists on the association of OPPs exposure with OA, except for one study that reported detectable serum concentrations of several pesticides, including OPPs, in a population with osteoarthritis [18]. Previous studies have reported that OPPs trigger immunosuppression/dysregulation through different mechanisms, thereby increasing host susceptibility to disease and a range of stressful conditions [49]. OPPs, such as chlorpyrifos and malathion, induce the release of pro-inflammatory cytokines such as IL-1β, IL-6, TNF-α and INF-γ. These cytokines are responsible for the activation and reassembly of nuclear transcription factors such as NF-κB and are involved in inflammation and apoptosis of damaged cells, ultimately lead to inflammatory changes in target organs related to OPPs (such as lungs, nerves, liver, tongue, etc.) [50–53]. Considering that pro-inflammatory cytokines, one of the key mediators of the pathophysiological disordered process of OA, especially IL-1β and TNF-α, control the degeneration of the articular cartilage matrix [54], it is reasonable to hypothesize that OPPs-mediated secretion of inflammatory mediators may be crucial for the development of OA. Although there is no direct evidence from previous studies indicating that OPPs have an impact on joint tissue, the mechanisms of OPPs in other diseases can interpret the link between OA and OPPs exposure observed in this study and may be a direction for further investigate.

Notably, one of the identified important risk factors for osteoarthritis is obesity [55, 56]. Epidemiologic investigations have shown that chronic exposure to organophosphorus pesticides is associated with increased waist circumference, body mass index, and prevalence of diabetes [57, 58]. Several ex vivo and in vivo experimental studies have also confirmed that OPPs affect glucose and lipid metabolic homeostasis, mainly in the form of hypertriglycerides, hyperglycemia, and hyperinsulinemia [59]. Whereas abnormalities in glucose and lipid metabolism are widely present in ASCVD patients [60], our study also reported that ASCVD patients had higher body mass index, triglyceride, and glycated hemoglobin levels compared to the non-ASCVD population. Therefore, we hypothesize that ASCVD patients with pre-existing glycolipid metabolism problems, such as obesity, are further affected by exposure to OPPs, increasing the frequency of OA. This metabolic factor may be one of the reasons why ASCVD patients are more susceptible to the arthrotoxicity of OPPs compared to the general population, and more in-depth studies are expected to provide more supporting evidence in the future.

The main strength of this study is the combination of multiple statistical methods to investigate the potential impact of exposure to OPPs (alone and mixed) on OA, which could enhance the robustness of our results. As far as we know, this is the first attempt to conduct a large-scale epidemiologic study to examine the relationship between OPPs exposure and OA (both in the general population and in the ASCVD population). Our findings are instructive for follow-up studies and emphasize the need for in vivo and in vitro experiments to elucidate underlying biological mechanisms. However, several limitations of this study should be noted. First, the nature of the cross-sectional design did not allow us to infer a causal relationship between open exposure and OA. In addition, the dialkyl phosphate (DAP) metabolites of OPPs are nonspecific, and we cannot know exactly which OPPs are the most harmful and need to be restricted for daily life use. Subsequent studies should therefore focus on developing measurements of specific biomarkers. Finally, despite adjusting for previously identified confounders in our statistical model, unmeasured or unknown factors may still play a non-negligible role in the association of OPPs exposure with OA.

## Conclusion

The current study suggests a possible link between exposure to OPPs (especially DMP and DEP) and an increased prevalence of OA in ASCVD patients. We also provide suggestive evidence for the cumulative effect of OPPs mixtures on OA, which predicts that ASCVD patients should be particularly careful in preventing the arthrotoxicity of OPPs. Further large-scale cohort studies and researches into the biological mechanisms are needed to confirm and clarify our findings.

### Electronic supplementary material

Below is the link to the electronic supplementary material.

Supplementary Material 1

Supplementary Material 2

Supplementary Material 3

Supplementary Material 4

Supplementary Material 5

Supplementary Material 6

## Data Availability

The datasets generated and analysed during the current study are available online in the NHANES repository [https://wwwn.cdc.gov/nchs/nhanes].
