# Supplementary figures and images for: Association between organophosphorus insecticides exposure and osteoarthritis in patients with arteriosclerotic cardiovascular disease
Source: BMC Public Health. 2024 Jul 14;24:1873. doi: 10.1186/s12889-024-19414-9 (PMC11247838; doi:10.1186/s12889-024-19414-9)

**Supplementary Figure S1.** Flowchart of participant selection process.


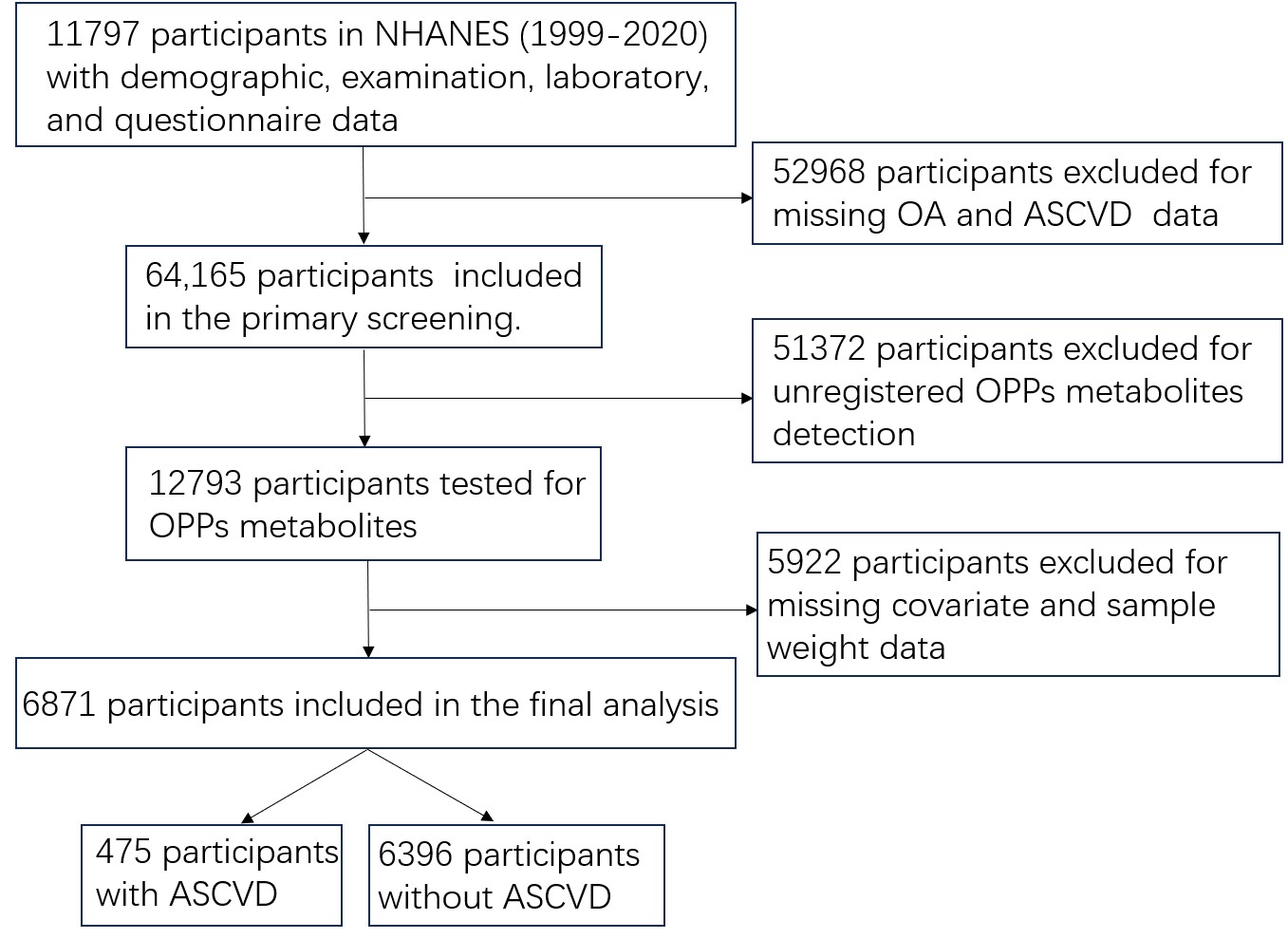

Supplement: Supplementary file 6 — Supplementary Material 6 [file 12889_2024_19414_MOESM6_ESM.docx]
